# The Legacy of the COVID-19 Pandemic: Impact on Infant and Maternal and Health from an Appalachian Academic Medical Center

**DOI:** 10.3390/children11080924

**Published:** 2024-07-30

**Authors:** Kelsey Haarbauer, Rebecca Burke, M. Cody Smith, Audrey N. Miller, Patricia N. Moran, Alicia A. Moise, Lesley Cottrell, Mark J. Polak

**Affiliations:** 1 Department of Pediatrics, West Virginia University School of Medicine, Morgantown, WV 26506, USA; kelsey.haarbauer@hsc.wvu.edu (K.H.); cody.smith1@hsc.wvu.edu (M.C.S.); lcottrell@hsc.wvu.edu (L.C.); 2 Division of Neonatology, Department of Pediatrics, Penn State College of Medicine, Penn State Health Children’s Hospital, Hershey, PA 17033, USA; rburke2@pennstatehealth.psu.edu; 3 Nationwide Children’s Hospital, Columbus, OH 43205, USA; audrey.miller@nationwidecholdrens.org; 4 Perinatal Medicine, Department of Pediatrics, University of Virginia, Charlottesville, VA 22903, USA; cgr6rt@uvahealth.org; 5 Department of Pediatrics, University of Oklahoma College of Medicine, Oklahoma City, OK 73104, USA; alicia.moise@ouhealth.com

**Keywords:** pre-pandemic period, COVID-19 pandemic, post-pandemic period, maternal health, infant health

## Abstract

Background/Objectives: The COVID-19 pandemic period from 2020 to 2022 had a significant impact on maternal infant health with mothers impacted more than their infants. We questioned whether there have been any lingering effects from the pandemic. Methods: We examined intermediate and long-term pandemic effects on maternal and neonatal outcomes before, during, and after the COVID-19 pandemic period. We reviewed mother–infant pairs from the following three epochs: (1) the pre–COVID-19 period, (2) the COVID-19 pandemic period, and (3) the post-pandemic period. The Case Mix Index (CMI) for the neonates from all three epochs were detailed. Results: Post-pandemic, we noted a rising trend of LGA infants (10%) and an increase in SGA infants (13%). For women in 2023, we noted an increase in hypertension, preeclampsia, diabetes, and a higher BMI than in the pre-pandemic period. There have also been more congenital anomalies (9%), and neonatal CMI increased in the post-pandemic period. Conclusions: Well after the pandemic period, maternal–infant health continues to be affected. For women, the increase in hypertension and diabetes during pregnancy is concerning. For infants, being LGA or SGA may have long-term consequences. The post-pandemic increase in infants with congenital anomalies compared to the pre-pandemic era is an area that needs ongoing review.

## 1. Introduction

The COVID-19 pandemic undoubtedly greatly impacted the lives and health of many individuals worldwide, some of which we are only beginning to discover and understand. This was particularly true as it pertained to pregnant mothers diagnosed with the virus and the effects that this may have had on their fetuses. Evidence in professional journals worldwide indicated that COVID-19 infection during pregnancy was associated with adverse pregnancy outcomes, especially among pregnant persons with infection acquired at early gestational ages, with a need for oxygen therapy, and with more symptomatic presentation [1]. Both higher and lower rates of preterm birth (earlier than 37 weeks gestation) have been reported across the literature, in addition to higher rates of preeclampsia, cesarean delivery, and stillbirth [2,3,4,5,6]. Studies also suggested that maternal COVID-19 infection was associated with neonatal morbidities such as hyperbilirubinemia, respiratory distress syndrome, and other neonatal respiratory disorders [5]. 

Gestational diabetes has also been reported as a pregnancy-associated complication of COVID-19 infection among mothers [7]. One study reported an increase in infants born large for gestational age (LGA) to mothers with gestational diabetes during the COVID-19 pandemic, even though there was no deterioration in prenatal care [8]. Infants who are born LGA or small for gestational age (SGA) often have increased short- and long-term adverse outcomes. Infants born LGA are at risk for neonatal intensive care admission, hypoglycemia, respiratory distress, and birth trauma [9]. Infants born SGA are at risk for prematurity-associated complications, adverse developmental outcomes, and changes to long-term adult health outcomes [10,11,12,13]. Given the significant impact on short- and long-term outcomes, we sought to determine if the COVID-19 pandemic impacted our infants’ outcomes, including LGA and SGA status. 

Our hospital, an Appalachian tertiary care center in Northeast West Virginia, did not admit its first pregnant mother with a known COVID-19 infection until mid-September 2020, several months after some other areas of the country and well after stay-at-home orders were issued in March 2020. During the height of the pandemic period (2020–2022), 8.2% of the mothers who delivered at our institution had been infected by COVID-19. For the infants, the most striking finding was an increase in large for gestation (LGA) infants to 13% and a decrease in small for gestation (SGA) infants to 3% [14].

As we moved past the pandemic, we questioned whether there had been any lingering effects from the pandemic regarding newborn and maternal health. 

## 2. Materials and Methods

After IRB approval, we conducted a chart review of infants and their mothers from three epochs. Mother–infant pairs delivered at the West Virginia University School of Medicine Ruby Memorial Hospital before the pandemic between May and July 2018 and April and June 2019 (*n* = 300 infants from 284 mothers) were reviewed. We examined the maternal–infant pairs with births during the height of the COVID-19 pandemic between 14 November 2020, and 30 April 2022. Mother–infant pairs from the COVID-19 pandemic were divided into the following two groups: infants born to mothers with COVID-19 infection during pregnancy (*n* = 305 infants from 298 mothers) and infants born to mothers without infection (*n* = 300 infants from 288 mothers). Finally, we reviewed mother–infant pairs from the 2023 post-pandemic period (*n* = 300 infants from 289 mothers).

We used Cogito Slicer Dicer, a self-service reporting tool with the Epic-electronic medical record system (Epic Systems, Verona, WI). Using this tool, we could identify the number of babies born and import the infants to appropriate data spreadsheets. We had access to critical criteria such as ICD-10 codes to filter maternal and infant comorbidities and characteristics. 

Infant delivery history, postnatal treatment during the infant hospitalization, and short-term health outcomes were extracted. For all infants, the Case Mix index (CMI) is derived from a modification of Medical Severity–Diagnosis Related Group (MS-DRG) weights, which we refer to as a Research-DRG (Table 1).

MS-DRG weights, rounded to a single decimal place determined by the United States Center for Medicare Services in 2023 [15], were used for all epochs. Differing from MS-DRG weights and the hospital CMI assignment, the weight of Research-DRG 789 (a weight of 1.8) was added to the assigned Research-DRG weight for any babies that died. The Research-DRG weight for every infant reviewed was assigned by a single investigator (MJP) using the criteria developed by Joya et al. [16]. 

**Table 1:** Description of Medical Severity–Diagnoses Related Groups (MS-DRG) with weight assigned and the DRG weight rounded for our research purposes (Research-DRG) adapted from previous work by Joya et al. [16]. All newborns were reviewed by author MJP, and a Research-DRG weight was assigned. Differing from hospital MS-DRG assignment, the weight of Research-DRG 789 (1.8) was added to the assigned Research-DRG weight for babies that died.

MS-DRG	Description	MS-DRG Weight	Research-DRG Weight
795	Normal newborn	0.2024	0.2
794	Newborn with significant problems	1.4946	1.5
793	Newborn with major problems	4.2225	4.2
792	Preterm newborn without major problems	2.4804	2.5
791	Preterm newborn with major problems	4.1107	4.1
790	Preterm less than 26 week gestation or respiratory distress syndrome (surfactant deficiency)	6.0189	6.0
789	Newborn less than 28 days that died or was transferred to another acute care facility	1.8252	1.8

For all mothers, several maternal comorbidities were extracted, including age, survival, presence of all hypertension (gestational and chronic hypertension, preeclampsia, and HELLP syndrome), diabetes (gestational, type 1, and type 2), body mass index (BMI), tobacco, drug use, and hepatitis C infection. For the second and third epochs, we also extracted the presence of COVID-19 infection, whether symptoms were noted, and whether any COVID-19–related treatment was provided. For mothers delivering after 15 April 2022 (the date that the vaccine was made available for low-risk adults), we documented COVID-19 vaccination status.

A total of 1205 infants were born to 1159 mothers at our institution during the three epochs (four groups). Comparative samples across four groups (~300 dyads) were utilized in this study. Before study planning, we calculated sample size based on assumptions including a medium effect size, significance level at *p* < 0.05, and power at 0.80. To adequately power the proposed analyses for this study, we needed to examine 223 dyads (G*power) [17]. Available records exceeded that sample size, and a cap of 300–305 was applied to each cohort. Descriptive analyses were conducted to characterize the four cohorts detailed in Table 2 and Table 3 and to explore the normative distribution of all information collected. Differences in infant and maternal outcomes and comorbidities were examined using one-way ANOVA for multiple comparisons. Statistical significance was established at the *p* < 0.05 level. SPSS version 28.0 was used to complete the analyses.

## 3. Results

Infant and maternal findings for all epochs are detailed in Table 2 and Table 3. 

Prior to the pandemic, our sample of 300 infants showed characteristics typical of babies born at a tertiary academic delivery service. The C-section rate was 37%, higher than the West Virginia state average of 34% [18]. However, we did not delineate primary versus repeat C-sections. Of babies born, 29% required NICU admission, which is higher than the national average of 9–13% [19], although likely similar to other tertiary NICUs associated with a high-risk delivery service. The percentage of babies born LGA and SGA was similar to regional averages at 7–8% [14]. Congenital anomalies ranged from congenital small head, hypospadias, and low-grade hydronephrosis to complex congenital heart disease. The remaining findings are consistent with findings from a tertiary delivery service. 

Reviewing all epochs, NICU admission rate, hypoglycemia, and RDS [ICD-10 = P22.0] was consistent across all epochs. Maternal opiate use with resulting neonatal opiate withdrawal syndrome (NOWS) is a chronic problem in Appalachia. However, our rate of 2–3% is lower than that in other parts of our state of West Virginia, which has been noted to be 5% [20]. Foster care of 3–5% is much higher than that noted in the United States [21]. 

During the pandemic period, whether or not mothers were infected, the infants seemed little affected in terms of acute medical comorbidities. Significant changes were most apparent in infants born to mothers free from COVID-19 infections during pregnancy. We noted a significant increase in instrumented vaginal deliveries. Of more importance, the percentage of LGA infants significantly increased (6% to 13%), and the percentage of SGA infants significantly decreased (7% to 4%). Curiously, we saw fewer preterm infants born to both groups of pandemic period mothers. This is similar to the reduction in preterm births noted globally [6]. The percentage of infants with any congenital anomalies was significantly lower in babies born to noninfected mothers during the pandemic, which we cannot easily explain. 

During the COVID-19 pandemic period, 288 women who had been diagnosed with COVID-19 during their pregnancies delivered babies at our institution, representing 8.2% of deliveries (305/3701). Overall, 59% of the women had symptoms ranging from mild cold–like nasal congestion to cardiorespiratory failure, with five women needing ECMO support and two of the women dying. Their diagnosis was made 57 ± 46 days prior to delivery, and 39% of their infants required NICU care. For asymptomatic mothers, their diagnosis was made 30 ± 57 days prior to delivery, with 8% of these asymptomatic women diagnosed upon admission to the labor and delivery service. Only 16% of the infants born to these mothers required NICU care. During this pandemic period, for mothers infected with COVID-19, the percentage presenting with hypertension showed a trend toward an increase, while all types of diabetes increased significantly.

For mothers infected with COVID-19 during pregnancy, whether or not they had symptoms, the short-term outcome for their infants was similar for all comorbidities (Table 4) with the exception of a higher percentage of hypoglycemia and the need for intravenous glucose in infants born to mothers symptomatic from the infection. This may be related to the increased percentage of diabetes noted in mothers infected with COVID-19. However, if we focus only on the infants born to mothers with severe disease (respiratory failure, needing oxygen, CPAP, ventilator support, or ECMO) we noted the following important clinical outcomes on infant health: 100% preterm, all requiring NICU admission, 86% needing C-sections, length of hospital stay nearly a month, 86% with hypoglycemia, and 71% with RDS (surfactant deficiency). Curiously, we noted 14% LGA babies consistent with our previously reported findings [14]. 

During 2023, representing the post-pandemic period, preterm births increased modestly. Instrumented vaginal deliveries have returned to pre-pandemic rates (1%). The most significant changes are continued increases in the percentages of both SGA (13%) and LGA infants (10%). The percentage of LGA births remained higher than pre-pandemic at 10%. The percentage of SGA infants increased to 13% from 4% during the pandemic.

**Figure 1:**
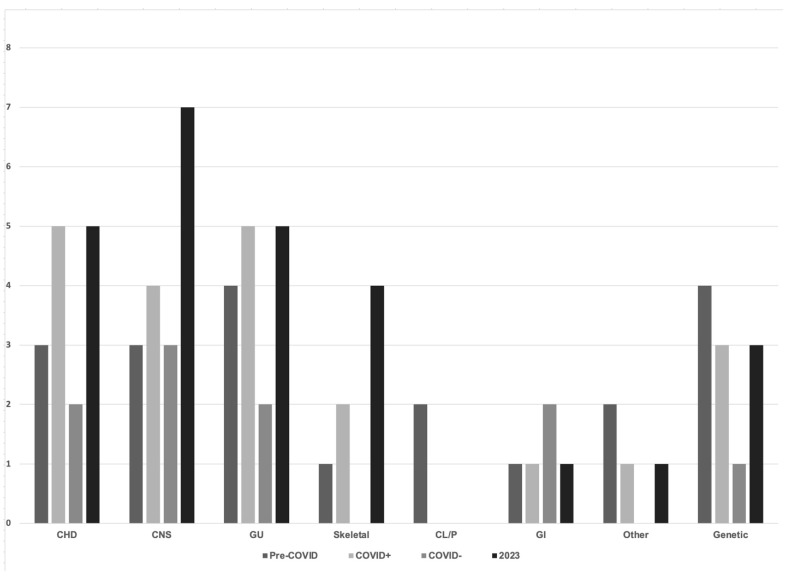
Congenital anomalies detected in newborns before, during, and after the COVID-19 pandemic. CHD—congenital heart disease; CNS—central nervous system defects; GU—genital urinary defects; Skeletal—spine and long bone anomalies; CL/P—cleft lip and palate; GI—gastrointestinal anomalies, defects; Other—nonspecific anomalies and defects not otherwise classified; Genetic—specific chromosomal anomalies or syndromes.

Congenital anomalies ranged from sacral dimples to lethal, complex congenital heart disease (Figure 1).

The percentage of infants with congenital anomalies was between 3% and 8% across all epochs and groups. We separately noted the incidence of congenital small head (a head circumference less than 3rd percentile for gestational age). Across all epochs, this remained stable at 1%. The percentage of infants with any congenital anomalies was significantly higher in babies born to infected mothers during the pandemic. Congenital heart disease, anomalies of the central nervous system, and genitourinary anomalies were the most frequently observed differences in infants across all epochs. The incidence of all congenital anomalies is not significantly different post-pandemic from those observed in the pre-pandemic period.

**Figure 2:**
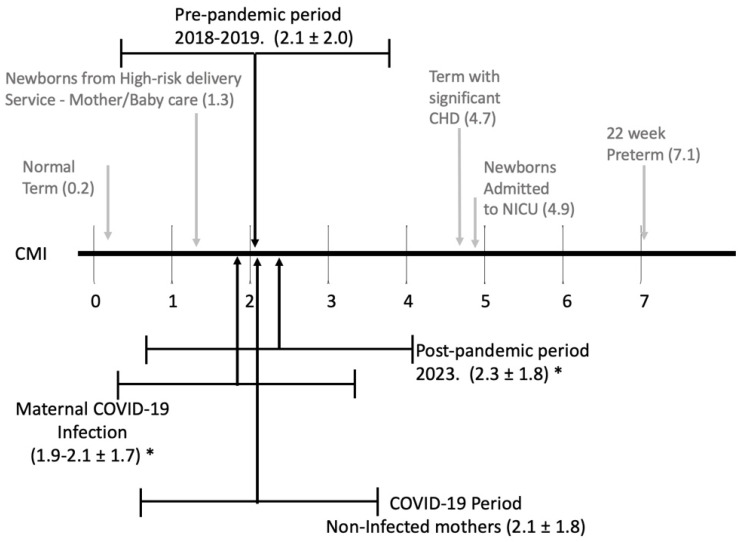
Case Mix Index (CMI) chart comparing the spectrum of neonatal care for newborns delivered at a high-risk, academic medical center, ranging from a normal newborn with 100% survival (Research-DRG weight = 0.2) to 22-week preterm, 30–35% survival (Research-DRG weight = 7.1) noted in gray. Chart adapted from previous work by Joya et al. [16]. In black are the average CMI for infants born at the same institution from the four epochs. * indicates a significant difference (*p* < 0.05) comparing CMI from infants born to mothers who had COVID-19 infection during pregnancy with infants born in the post-pandemic period of 2023.

Figure 2 details the Case Mix Index (CMI) for each epoch compared to the spectrum of CMI for infants, with a breakdown of the research DRGs and weights noted in Table 5.

The CMI in the post-pandemic period was significantly higher than the CMI from the pandemic period. This is likely related to significantly more late preterm and term infants needing respiratory support (NCPAP and/or ventilator support) and more term infants with at least one significant problem (infants affected by maternal conditions, medications, or congenital anomalies). We also noted a significant decrease in normal-term infants and a trend to fewer preterm infants without major problems.

The Appalachian region of the United States, and especially the state of West Virginia, has consistently ranked low for key health indicators for adults, for substance use, and for drug overdose deaths. This is certainly evident in the maternal data from all epochs. The average BMI ranged from 33.3 to 34.8 kg/m^2^, with 61% to 70% of these women delivering with a BMI greater than 30 kg/m^2^, higher than the US average of 40% [22]. During the pre-pandemic period, the percentage of diabetes and maternal hypertension was higher than that noted in the country as a whole [22,23]. Across all epochs, tobacco use was twice that of the rest of the country [24]. Illicit substance use (opiates, cannabis, cocaine, and methamphetamine) was similar to that noted throughout the United States [25]. 

During 2023 (the post-pandemic period), the most striking changes were a significant increase in hypertension (including preeclampsia and HELLP syndrome) and a trend toward more mothers with diabetes. Also, perinatal COVID-19 infections continued to occur at a rate of approximately 4% of women, although the degree of illness was subjectively lower than during the pandemic period. Maternal utilization of COVID-19 vaccines was 43%, similar to that of pregnant women throughout the country, although lower than the population as a whole, which is close to 78% [26].

## 4. Discussion

The increase in LGA infants was first noted by parents during the pandemic period and shared through social media platforms with multiple videos [27]. Subsequent review of our data showed that there was a significant increase in LGA infants and a significant decrease in SGA infants being the major finding [14], along with a trend toward more congenital anomalies. As the introduction of this paper details, being born large or small for gestational age may have long-term consequences [7,8,9,10,11,12,13]. Barker hypothesized that SGA infants have a high incidence of coronary heart disease, diabetes mellitus, hyperinsulinemia, and hypercholesterolemia as adults [28]. LGA infants are at an increased risk of becoming overweight and obese later in life compared to their normal-weight counterparts. Infants born greater than 4000 g have a 50% increased risk of becoming overweight later in life. LGA infants over 4500 g have a 19% increase in the risk of developing type 2 diabetes mellitus as an adult compared to those with birth weights between 4000 and 4500 g [28].

The NICU admission rate ranged from 23% to 30%, which is consistent with a high-risk delivery service, as are the percentages of RDS [ICD-10: P22.0] between 7% and 11% as well as the overall percentage of infants born prior to 37 weeks at 19–28% compared to the national average of 12% [19]. The rates of neonatal hypoglycemia from our institution ranged from 16% to 21%, which is higher than that noted for US infants, reported as 5–15% [29]. NOWS ranged from 2% to 5%, with the national average of 1.2% of Medicaid-eligible infants [25]. The need for foster care at 3–5% is the highest in the country, compared to 1.3% nationally [21,21].

Early in the pandemic, there was concern regarding the possibility of teratogenic effects of the mRNA vaccine on the developing fetus during critical windows of organogenesis. Several authors have explored this possibility and found no composite increase in anomalies in infants born to mothers who received the vaccine during the first trimester versus those who did not [30,31,32]. Additional reports about vaccine safety during the teratogenic window of gestation only considered congenital anomalies detected via ultrasonography rather than neonatal outcomes [33], allowing the possibility that more subtle anomalies not identified on an ultrasound could have been missed. In our cohort during the period considered to be the COVID-19 pandemic (from September 2020 to April 2022), significantly fewer infants were born with congenital anomalies to mothers not infected with COVID-19. Vaccination status was not considered in this cohort, but rather actual COVID-19 infection. The etiology of this phenomenon of increased congenital anomalies in COVID-19–infected mothers is unclear. Considerations as to the etiology of anomalies in infants born to COVID-19–infected mothers in our cohort include maternal hyperthermia, which has been associated with an increase in congenital anomalies of the central nervous system such as neural tube defects, gastroschisis, and cardiac anomalies [34,35,36,37]. A cohort in Iran reported a similar phenomenon where CNS and genitourinary anomalies significantly increased during the COVID-19 pandemic [38]. This group also speculates maternal hyperthermia to be potentially causative in addition to vertical transmission of COVID-19 infection, stress and anxiety, insufficient preconception and prenatal care, neglect of fetal screening, and poverty imposed by this pandemic. These additional factors are undoubtedly relevant to our rural, resource-limited Appalachian population. A limitation for our cohort is that the timing of COVID-19 infection is not known in the mothers. Thus, whether hyperthermia was likely to occur during the first trimester is unknown. A true TORCH-like or intrinsic teratogenic effect of COVID-19 seems less likely, given the paucity of reports on increases in congenital anomalies during the COVID-19 pandemic. The effect of the hypercoagulable state known to result from maternal COVID-19 infection was considered by Repucci et al. as a possible cause for congenital anomalies secondary to in utero vascular accidents and found not to be associated with GI or limb anomalies in their relatively small single-center American cohort [39]. Additionally, increases in microcephaly noted in the late pandemic period in a Canadian cohort was concluded to be more likely secondary to an artifact of enhanced surveillance for anomalies [40], and no conclusion could be made as to increases in GI anomalies due to overall low incidence in a Romanian cohort [41].

We have previously reported that the percentage of infants with microcephaly, defined as a head circumference less than 3% for gestational age, is higher than the national average (1.02% versus 0.5% reported for the nation) [42]. In a review of the documented congenital anomalies, the microcephaly rate remained at 1% throughout all epics. We postulated that the combination of tobacco and illicit substance use was a factor in this higher rate of microcephaly and believe this is still a significant factor.

The health concerns for women of childbearing age (14–44 years) in Appalachia have been well documented. The rates of obesity, tobacco, drug use, diabetes, and hypertension are all above national averages [43]. Our sample population prior to the pandemic reflects these concerns. Despite this, the infants born in our academic referral hospital seem to fit within national averages.

The American College of Obstetricians and Gynecologists (ACOG) considers a BMI > 30 kg/m^2^ as obese, with 40% of pregnant women in the United States considered obese [22]. From all epochs, 61–70% of the mothers had a BMI greater than 30 kg/m^2^, with >80% greater than 30 kg/m^2^ for mothers of LGA babies. The C-section rate of 37–39% is higher than the national average of 20–32%, although we did not differentiate between primary and repeat C-section [44]. All types of diabetes at 7–13% are higher than the national average of 1–2%. There is increasing evidence that suggests that intrauterine hyperglycemia may disrupt the neurocircuitry of the fetus, which may predispose the children to the development of neurocognitive and behavioral problems. This likely occurs through the induction of an exaggerated peripheral inflammatory response and neuroinflammation in the fetal brain [45]. 

Between 21% and 38% of pregnant women develop hypertension, with a significant increase noted post-pandemic. These rates are higher than the national averages of 8–16%. Women with chronic hypertension have a higher incidence of superimposed preeclampsia, C-section, preterm delivery before 37 weeks gestation, birth weight less than 2500 g, neonatal unit admission, and perinatal death. They also have a higher risk of developing cardiovascular disease later in life [46]. The significant increase in babies born small for gestational age likely mirrors the significant increase in mothers with hypertension during pregnancy. We have found that the rate of maternal hypertension in the post-pandemic epoch has continued to show significant increases. From 21% in the pre-pandemic epoch to 32% during the pandemic, and 38% of pregnant women are diagnosed with hypertension in the post-pandemic period. Some studies now suggest that COVID-19 may have a lasting impact on the cardiovascular system for some, with an increase seen in the development of either new onset hypertension or a worsening of existing hypertensive conditions [47,48,49]. It remains unclear and a topic for further study whether or not these complications will resolve over time or have a lasting effect. It is also unclear why effects occur, whether it is a direct effect of the virus itself or whether the indirect effects of the pandemic, including social isolation, poor diet, physical activity, weight gain, psychosocial stress, and others, also play a role. We are unable to delineate with certainty which of the pregnant mothers in our post-pandemic study population ever had a prior COVID-19 infection, which may be contributing to the increase in hypertension but may be a topic for further investigation. It has been well-documented that hypertension over time is associated with and is a significant risk factor in the development of various cardiovascular diseases and chronic kidney disease [50]. Maternal hypertension and hypertensive disorders are also highly detrimental to the fetus, as it is a leading cause of prematurity, abnormal birth weight (either high or low for gestational age), and mortality and are being shown to put these individuals at risk for cardiovascular disease, hypertension, and metabolic disease later in life [51,52]. This alarming trend that impacts both the mother and the fetus deserves further attention going forward. 

Tobacco use by pregnant women in the state of West Virginia is the highest in the country at approximately 23%. However, the rate from our institution is lower, ranging from 13% to 16% overall in all epochs, with as low as 9% (COVID-19 symptomatic mothers) and as high as 43% (COVID-19 pandemic period delivering SGA infants) [14]. The prevalence of maternal smoking at any time during pregnancy in the United States is 7.2% [41]. Throughout all epochs, illicit substance use of opiates, cannabis, cocaine, and methamphetamine was similar and in line with national averages [53]. 

Of the mothers with pandemic era COVID-19 infections, 59% were symptomatic, with five progressing to ECMO and two dying after the delivery of their infants. Overall, the infants born to mothers symptomatic from the infection did well, although if mother had severe disease, all infants were born preterm, needed NICU care, 71% had RDS (surfactant deficiency), and 86% had hypoglycemia. In the post-pandemic period, perinatal COVID-19 infections were noted in about 4% of mothers giving birth. We still found that 50% showed symptoms, although none have required hospitalizations. Maternal COVID-19 vaccination rates were 43%, similar to US vaccination rates for pregnant women, although this rate lags behind vaccination rates for the US population [54]. 

## 5. Conclusions

Our investigation reflects maternal infant health from a single academic, tertiary care medical center in the Appalachian region of the United States, which may be unique to our region. In the pre-pandemic period, our maternal/infant data mirrors that from the entire Appalachian region. In general, they are showing maternal health concerns in several areas. For infants, the impact of maternal substance use with NOWS and the need for foster placement is higher than national averages [55]. Although our ability to accurately track maternal COVID-19 infections came late to our region, it is clear that COVID-19 infections in women of childbearing age have been impactful. It is interesting to note that infants whose mothers were symptomatic had more than twice the rate of NICU admission as compared to those whose mothers were asymptomatic (39% vs. only 18%). Factors contributing to this discrepancy are an area that may be of interest to future investigations. Fortunately, the infants have, in the short term, seemed to weather their mother’s illnesses well and have had an extremely high survival rate thus far. We are continuing to monitor our infants exposed prenatally to COVID-19 as this infection continues to affect over 4% of mothers. It will be valuable to continue to follow these infants for any long-term health and developmental concerns. 

Well after the pandemic period, maternal–infant health continues to be affected. For our mothers, the increase in hypertension and diabetes during pregnancy is concerning. For infants, being large or small for gestational age may have long-term consequences. We remain concerned about the high percentage of infants with microcephaly across all epochs. The post-pandemic health changes in our mothers and infants may be unique to our Appalachian region, although we should be alert for healthcare workers dealing with individuals or groups that share similar characteristics in socioeconomic status, lifestyle, and limited access to basic healthcare needs. Moving forward, COVID-19 continues to mutate and infect our mothers. In the short term, babies seem unfazed. However, long-term follow-up has been showing changes in the gut microbiome [56] and there are concerns about some developmental delays, mainly speech and possibly gross motor [57].

## Figures and Tables

**Table 2:** Neonatal data from the four epochs.

	Pre-COVID 2018–2019 (*n* = 300)	COVID-19 Period Infected Mothers 2020–2022 (*n* = 305)	COVID-19 Period Noninfected Mothers 2020–2022 (*n* = 300)	Post COVID-19 2023 (*n* = 300)	*p* < 0.05
Gestation (weeks)	37 ± 3	38 ± 2	38 ± 3	37 ± 3	NS
Prematurity < 36 6/7 wk.	28%	19% *	19% *	23% *	0.011
Male	56%	53%	51%	53%	NS
C-section	37%	36%	39%	37%	NS
Instrumented vaginal deliveries	1% *	4%	8% *	1% *	0.014
LOS (days)	10 ± 24 *	7 ± 20	7 ± 18	6 ± 15 *	0.036
Apgar 1 min	7 ± 2	8 ± 2	8 ± 2	7 ± 2	NS
Apgar 5 min	9 ± 1	9 ± 1	9 ± 1	9 ± 1	NS
Z score for weight	−0.04 ± 0.83	0.07 ± 0.94	0.10 ± 0.92	0.04 ± 0.98	NS
Large for gestational age (LGA)	6% *	10%	13% *	10%	0.041
Small for gestational age (SGA)	7%	6%	4% *	13% *	0.02
Hypoglycemia	18%	16%	19%	18%	NS
Hypoglycemia +IV fluids	72.2%	62.5%	47.4%	50%	NS
NICU Admission	29%	23%	26%	30%	NS
RDS [P22.0]	11%	7%	8%	7%	NS
Neonatal COVID-19	-	0.6%	0	0	
Congenital anomaly	5%	7%	3% *	8% *	0.022
Microcephaly (<3% for gestational age)	1%	1%	0%	1%	NS
NOWS	2%	2%	3%	2%	NS
Foster care	5%	4%	3%	3%	NS
Twin	10%	8%	9%	6%	NS
Survival	99%	99.3%	99.7%	99%	NS

* indicated significant differences with *p* value indicated.

**Table 3:** Maternal data from the four epochs.

	Pre-COVID 2018–2019 (*n* = 284)	COVID-19 Period Infected Mothers 2020–2022 (*n* = 298)	COVID-19 Period Noninfected Mothers 2020–2022 (*n* = 288)	Post COVID-19 2023 (*n* = 289)	*p* < 0.05
Maternal age (years)	28 ± 6	27± 5	28 ± 5	28 ± 5	NS
Survival	100%	99%	100%	100%	NS
BMI	33.29 ± 14.39	34.85 ± 8.44	34.49 ± 8.12	34.57 ± 8.45	NS
BMI > 30	61%	70%	67%	69%	NS
Tobacco exposure	14%	13%	16%	15%	NS
Cannabis exposure	5%	3%	9%	7%	NS
Opioid exposure	6%	2%	6%	4%	NS
Cocaine exposure	2%	0	1%	0	NS
Methamphetamine exposure	1%	0	2%	2%	NS
Hepatitis C. positive	4%	3%	2%	4%	NS
Hypertension (+ preeclampsia and HELLP)	21% *	32%	32%	38% *	0.001
Diabetes (including GDM, type 1, and type 2)	7% *	13% *	12%	11%	0.02
COVID-19 infection	-	100%	-	4%	NS
Days from COVID-19 infection to delivery	-	66 ± 68	-	50–260	NS
Symptomatic COVID-19 infection	-	59%	-	50%	NS
COVID-19 antibody treatment	-	6.2%	-	10%	NS
NCPAP/vent support (without ECMO)	-	0.6%	-	0	NS
ECMO + vent support	-	1.7%	-	0	NS
COVID-19 vaccine (after 15 April 2021)	-	13%	14%	43%	NS

* indicated significant differences with p value indicated.

**Table 4:** Neonatal data: Infants born to asymptomatic versus symptomatic mothers. A subset of babies born to mothers with severe disease is included. Neonatal comorbidities from asymptomatic versus all symptomatic mothers is compared by ANOVA.

Maternal COVID-19 Infections	COVID-19 Asymptomatic (*n* = 120)	COVID-19 Symptomatic (*n* = 185)	Significance *p* ≤ 0.05	COVID-19 Severe Symptoms (*n* = 7)
Gestation (weeks)	38 ± 2	38 ± 2	NS	32 ± 3
Prematurity < 36 6/7 wk.	18%	20%	NS	100%
Male	55%	50%	NS	57
C-section	31%	41%	NS	86%
Instrumented vaginal deliveries	4%	4%	NS	0
LOS (days)	6 ± 18	7 ± 21	NS	27 ± 25
Apgar 1 minute	8 ± 2	7 ± 2	NS	6 ± 2
Apgar 5 minute	9 ± 1	9 ± 1	NS	8 ± 1
Z score for weight	0.00 ± 0.93	0.13 ± 0.89	NS	0.73 ± 0.36
Large for gestational age (LGA)	9%	11%	NS	14%
Small for gestational age (SGA)	7%	5%	NS	0
Hypoglycemia	13%	18%	NS	86%
Hypoglycemia + IV fluids	46%	66.6%	*p* = 0.05	74%
NICU Admission	19%	26%	*p* = 0.05	100%
RDS [P22.0]	4%	8%	NS	71%
Neonatal COVID-19	0%	0.5%	NS	0
Congenital anomaly	6%	8%	NS	0
NALS/NOWS	3%	1%	NS	0
Survival	99.2%	99%	NS	100%

**Table 5:** Case Mix Index (CMI) and breakdown of research DRGs assigned for all four epochs. Percentages for Research-DRG 789 indicates neonatal deaths from each epoch. *, §, and ¶ indicate significant (*p* < 0.05) difference of the research-DRGs between epochs.

	Pre-COVID 2018–2019 (*n* = 300)	COVID-19 Period Infected Mothers 2020–2022 (*n* = 305)	COVID-19 Period Noninfected Mothers 2020–2022 (*n* = 300)	Post COVID-19 2023 (*n* = 300)
Case Mix Index CMI	2.1 ± 2.0	1.9 ± 1.7 *	2.1± 1.8	2.3 ± 1.8 *
Research-DRG 795 Weight = 0.2	35% *	31%	28%	22% *
Research-DRG 794 Weight = 1.5	28% * § ¶	42% §	39% ¶	42% *
Research-DRG 792 Weight = 2.5	8%	9%	4%	4%
Research-DRG 791 Weight = 4.1	10%	6% §	7%	14% * §
Research-DRG 793 Weight = 4.2	9% *	9% §	13%	16% * §
Research-DRG 790 Weight = 6.0	10%	7%	8%	7%
Research-DRG 789 Weight = 1.8	1%	0.7%	0.3%	0.7%

## Data Availability

At this time, the patient data is unavailable due to privacy or ethical restrictions (HIPAA).

## References

[1] Dubey P., Reddy S.Y., Manuel S., Dwivedi A.K. (2020). Maternal and neonatal characteristics and outcomes among COVID-19 infected women: An updated systematic review and meta-analysis. EJOG.

[2] Di Mascio D., Khalil A., Saccone G., Rizzo G., Buca D., Liberati M., Vecchiet J., Nappi L., Scambia G., Berghella V. (2020). Outcome of coronavirus spectrum infections (SARS, MERS, COVID-19) during pregnancy: A systematic review and meta-analysis. AJOG MFM.

[3] Novoa R.H., Quintana W., Llancarí P., Urbina-Quispe K., Guevara-Ríos E., Ventura W. (2021). Maternal clinical Characteristics, and perinatal outcomes among pregnant women with coronavirus disease 2019. A systematic review. Travel Med. Infect. Di..

[4] Woodworth K.R. (2020). Birth and Infant Outcomes Following Laboratory-Confirmed SARS-CoV-2 Infection in Pregnancy—SET-NET, 16 Jurisdictions, March 29–October 14, 2020. MMWR.

[5] Norman M., Navér L., Söderling J., Ahlberg M., Hervius Askling H., Aronsson B., Byström E., Jonsson J., Sengpiel V., Ludvigsson J.F. (2021). Association of Maternal SARS-CoV-2 Infection in Pregnancy with Neonatal Outcomes. JAMA.

[6] Calvert C., Brockway M., Zoega H., Miller J.E., Been J.V., Amegah A.K., Racine-Poon A., Oskoui S.E., Abok I.I., Aghaeepour N. (2023). Changes in preterm birth and stillbirth during COVID-19 lockdowns in 26 countries. Nat. Hum. Behav..

[7] Auger N., Wei S.Q., Dayan N., Ukah U.V., Quach C., Lewin A., Healy-Profitós J., Ayoub A., Chang J., Luu T.M. (2023). Impact of COVID-19 on rates of gestational diabetes in a North American pandemic epicenter. Acta Diabetol..

[8] Vicente D.M.C., Martínez A.M., García I.G., Toboso R.Q., López I.Q., Rey M.D., Vaamonde J.G., Alemán M.O., Moragrega R.M., Díaz C.G. (2024). Effects of the COVID-19 pandemic on gestational diabetes in Castilla-La Mancha (Spain). Endocrinol. Diabetes Nutr..

[9] Scifres C.M. (2021). Short- and Long-Term Outcomes Associated with Large for Gestational Age Birth Weight. Obstet. Gynecol. Clin. North. Am..

[10] Charles E., Hunt K.A., Harris C., Hickey A., Greenough A. (2019). Small for gestational age and extremely low birth weight infant outcomes. J. Perinat. Med..

[11] Campisi S.C., Carbone S.E., Zlotkin S. (2019). Catch-Up Growth in Full-Term Small for Gestational Age Infants: A Systematic Review. Adv. Nutr..

[12] Li X., Eiden R.D., Epstein L.H., Shenassa E.D., Xie C., Wen X. (2016). Etiological Subgroups of Small-for-Gestational-Age: Differential Neurodevelopmental Outcomes. PLoS ONE.

[13] Minor K.C., Bianco K., Sie L., Druzin M.L., Lee H.C., Leonard S.A. (2023). Severity of small-for-gestational-age and morbidity and mortality among very preterm neonates. J. Perinatol..

[14] Moran T., Moise A., Miller A., Burke R., Cottrell L., Haarbauer K., Smith M.C., Polak M. (2023). Changes in Large for Gestation and Small for Gestation Births during the COVID-19 Era. WVMJ.

[15] FY 2024 IPPS Proposed Rule Home Page. https://www.cms.gov/medicare/payment/prospective-payment-systems/acute-inpatient-pps/fy-2024-ipps-proposed-rule-home-page.

[16] Joya R.M., Cottrell L., Kiefer A., Polak M.J. (2022). Diagnosis Related Group Weight and Derived Case Mix Index to Assess the Complexity Among Twins. Am. J. Perinatol..

[17] Faul F., Erdfelder E., Buchner A. (2009). Statistical power analyses using G*Power 3.1, Tests for correlation and regression analyses. Behav. Res. Methods.

[18] https://www.cdc.gov/nchs/pressroom/states/westvirginia/wv.htm.

[19] Pineda R., Knudsen K., Breault C.C., Rogers E.E., Mack W.J., Fernandez-Fernandez A. (2023). NICUs in the US: Levels of acuity, Number of Beds, and Relationships to Population Factors. J. Perinatol..

[20] Umer A., Loudin S., Maxwell S., Lilly C., Stabler M.E., Cottrell L., Hamilton C., Breyel J., Mullins C., John C. (2019). Capturing the Statewide Incidence of Neonatal Abstinence Syndrome in Real Time: The West Virginia Experience. Ped Res..

[21] Grouse G., Ghertner R., Madden E., Radel L. (2021). Foster Care Entry Rates Grew Faster for Infants than for Children of Other Ages, 2011–2018. https://www.aspe.hhs.gov/sites/default/files/2021-08/infant-foster-care-brief.pdf.

[22] (2021). Committee on Practice Bulletins—Obstetrics. Obes. Pregnancy Obstet. Gynecol..

[23] Khedagi A.M., Bello N.A. (2021). Hypertensive Disorders of Pregnancy. Card. Clin..

[24] https://www.cdc.gov/diabetes/php/data-research/.

[25] Forray A. (2016). Substance Use during Pregnancy. F1000 Res..

[26] Regan A.K., Kaur R., Nosek M., Swathi P.A., Gu N.Y. (2022). COVID-19 vaccine acceptance and coverage among pregnant persons in the United States. Prev. Med. Rep..

[27] Loveandpositiveenergy (2021). Build like Warriors #Bigpandemicbabies. TikTok. www.tiktok.com.

[28] Barker D. (2002). Fetal programming of coronary heart disease. Trends Endocrinol. Metab..

[29] Edwards T., Harding J. (2021). Clinical Aspects of Neonatal Hypoglycemia: A Mini Review. Front. Pediatr..

[30] Woestenberg P.J., de Feijter M., Bergman J.E.H., Lutke L.R., Passier A.J.L.M., Kant A.C. (2023). Maternal first trimester COVID-19 vaccination and risk of major non-genetic congenital anomalies. Birth Defects Res..

[31] Santos J., Miller M., Branda M.E., Mehta R.A., Theiler R.N. (2024). Maternal COVID-19 vaccination status and association with neonatal congenital anomalies. Front. Pediatr..

[32] Ding C., Liu Y., Pang W., Zhang D., Wang K., Chen Y. (2023). Associations of COVID-19 vaccination during pregnancy with adverse neonatal and maternal outcomes: A systematic review and meta-analysis. Front. Public Health.

[33] Ruderman R.S., Mormol J., Trawick E., Perry M.F., Allen E.C., Millan D., Miller E.S. (2022). Association of COVID-19 Vaccination During Early Pregnancy with Risk of Congenital Fetal Anomalies. JAMA Pediatr..

[34] Luteijn J.M., Brown M.J., Dolk H. (2014). Influenza and congenital anomalies: A systematic review and meta-analysis. Hum. Reprod..

[35] Botto L.D., Panichello J.D., Browne M.L., Krikov S., Feldkamp M.L., Lammer E., Shaw G.M., National Birth Defects Prevention Study (2014). Congenital heart defects after maternal fever. Am. J. Obstet. Gynecol..

[36] Graham J.M. (2020). Update on the gestational effects of maternal hyperthermia. Birth Defects Res..

[37] Suarez L., Felkner M., Hendricks K. (2004). The effect of fever, febrile illnesses, and heat exposures on the risk of neural tube defects in a Texas-Mexico border population. Birth Defects Res. A Clin. Mol. Teratol..

[38] Heidarzadeh M., Taheri M., Mazaheripour Z., Abbasi-Khameneh F. (2022). The incidence of congenital anomalies in infants before and during the COVID-19 pandemic. Ital. J. Pediatr..

[39] Reppucci M.L., Kaizer A.M., Prendergast C., Acker S.N., Mandell E.W., Euser A.G., Diaz-Miron J. (2023). Incidence of congenital complications related to COVID-19 infection during pregnancy. J. Neonat. Perinat. Med..

[40] Auger N., Arbour L., Lewin A., Brousseau É., Healy-Profitós J., Luu T.M. (2024). Congenital anomalies during COVID-19: Artifact of surveillance or a real TORCH?. Eur. J. Epidemiol..

[41] Brandibur T.E., Kundnani N.R., Boia M., Nistor D., Velimirovici D.M., Mada L., Manea A.M., Boia E.R., Neagu M.N., Popoiu C.M. (2023). Does COVID-19 Infection during Pregnancy Increase the Appearance of Congenital Gastrointestinal Malformations in Neonates?. Biomedicines.

[42] Moore C.A., Moise A., Cottrell L., Polak M.J. (2023). Incidence of Congenital Microcephaly is Increased With Fetal Exposure to Tobacco, Opioids, and Cannabis. WVMJ.

[43] Pederson A. (2022). Maternal Mortality Rates in Appalachia. https://pitjournal.unc.edu/2023/03/22/maternal-mortality-rates-in-appalachia.

[44] Michelle J.K., Osterman M.H.S. Changes in Primary and Repeat Cesarean Delivery: United States, 2016–2021. https://www.cdc.gov/nchs/data/vsrr/vsrr021.pdf.

[45] Rodolaki K., Pergialiotis V., Iakovidou N., Boutsikou T., Iliodromiti Z., Kanaka-Gantenbein C. (2023). The impact of maternal diabetes on the future health and neurodevelopment of the offspring: A review of the evidence. Front. Endocrinol..

[46] Agrawal A., Wenger N.K. (2020). Hypertension During Pregnancy. Curr. Hypertens. Rep..

[47] Delalić Đ., Jug J., Prkačin I. (2022). Arterial Hypertension Following COVID-19, A Retrospective Study of Patients in a Central European Tertiary Care Center. Acta Clin. Croat..

[48] Zhang V., Fisher M., Hou W., Zhang L., Duong T.Q. (2023). Incidence of New-Onset Hypertension Post-COVID-19: Comparison With Influenza. Hypertension.

[49] Akpek M. (2022). Does COVID-19 Cause Hypertension?. Angiology.

[50] Fuchs F.D., Whelton P.K. (2020). High Blood Pressure and Cardiovascular Disease. Hypertension.

[51] Cunningham M.W., LaMarca B. (2018). Risk of cardiovascular disease, end-stage renal disease, and stroke in postpartum women and their fetuses after a hypertensive pregnancy. Am. J. Physiol. Regul. Integr. Comp. Physiol..

[52] Brown M.C., Best K.E., Pearce M.S., Waugh J., Robson S.C., Bell R. (2013). Cardiovascular disease risk in women with pre-eclampsia: Systematic review and meta-analysis. Eur. J. Epidemiol..

[53] About Opioid Use during Pregnancy. https://www.cdc.gov/pregnancy/opioids/data.

[54] Shephard H.M., Manning S.E., Nestoridi E., Darling A.M., Brown C.M., Hatch M., Ahnger-Pier K., Pagnano S., Mather D., Yazdy M.M. (2023). Inequities in COVID-19 Vaccination Coverage Among Pregnant Persons, by Disaggregated Race and Ethnicity—Massachusetts, May 2021–October 2022. MMWR.

[55] Births and Natality. https://www.cdc.gov/nchs/fastats/births.htm.

[56] Querdasi F.R., Vogel S.C., Thomason M.E., Callaghan B.L., Brito N.H. (2023). A comparison of the infant gut microbiome before versus after the start of the COVID-19 pandemic. Sci. Rep..

[57] Giesbrecht G.F.P., Lebel C., Dennis C.-L., Silang K.B., Xie E.B.M., Tough S., McDonald S., Tomfohr-Madsen L.P. (2023). Risk for Developmental Delay Among Infants Born During the COVID-19 Pandemic. JDBP.

